# Human DNA methylomes of neurodegenerative diseases show common epigenomic patterns

**DOI:** 10.1038/tp.2015.214

**Published:** 2016-01-19

**Authors:** J V Sanchez-Mut, H Heyn, E Vidal, S Moran, S Sayols, R Delgado-Morales, M D Schultz, B Ansoleaga, P Garcia-Esparcia, M Pons-Espinal, M M de Lagran, J Dopazo, A Rabano, J Avila, M Dierssen, I Lott, I Ferrer, J R Ecker, M Esteller

**Affiliations:** 1 Epigenetics and Biology Program, Bellvitge Biomedical Research Institute, Barcelona, Spain; 2 Genomic Analysis Laboratory, The Salk Institute for Biological Studies, La Jolla, CA, USA; 3 Bioinformatics Program, University of California at San Diego, La Jolla, CA, USA; 4 Neuropathology Institute, Bellvitge Biomedical Research Institute-Hospital Universitari de Bellvitge, Universitat de Barcelona, Centro de Investigación Biomédica en Red de Enfermedades Neurodegenerativas, Barcelona, Spain; 5 Centre for Genomic Regulation, Universitat Pompeu Fabra, Barcelona, Spain; 6 Centro de Investigación Biomédica en Red de Enfermedades Raras, Barcelona, Spain; 7 Computational Genomics Department, Centro de Investigación Príncipe Felipe, Valencia, Spain; 8 Neuropathology Laboratory, Research Unit Alzheimer's Project, Fundación CIEN, Madrid, Spain; 9 Department of Neuroscience, Centro de Biología Molecular Severo Ochoa CSIC/UAM, Universidad Autónoma de Madrid, Madrid, Spain; 10 Centro de Investigación Biomédica en Red de Enfermedades Neurodegenerativas, Madrid, Spain; 11 Department of Pediatrics and Neurology, School of Medicine, University of California, Irvine, Irvine, CA, USA; 12 Howard Hughes Medical Institute, The Salk Institute for Biological Studies, La Jolla, CA, USA; 13 Department of Physiological Sciences II, School of Medicine, University of Barcelona, Barcelona, Spain; 14 Institució Catalana de Recerca i Estudis Avançats, Barcelona, Spain

## Abstract

Different neurodegenerative disorders often show similar lesions, such as the presence of amyloid plaques, TAU-neurotangles and synuclein inclusions. The genetically inherited forms are rare, so we wondered whether shared epigenetic aberrations, such as those affecting DNA methylation, might also exist. The studied samples were gray matter samples from the prefrontal cortex of control and neurodegenerative disease-associated cases. We performed the DNA methylation analyses of Alzheimer's disease, dementia with Lewy bodies, Parkinson's disease and Alzheimer-like neurodegenerative profile associated with Down's syndrome samples. The DNA methylation landscapes obtained show that neurodegenerative diseases share similar aberrant CpG methylation shifts targeting a defined gene set. Our findings suggest that neurodegenerative disorders might have similar pathogenetic mechanisms that subsequently evolve into different clinical entities. The identified aberrant DNA methylation changes can be used as biomarkers of the disorders and as potential new targets for the development of new therapies.

## Introduction

Neurodegenerative diseases are complex disorders caused by the convergence of genetic and environmental factors in aging. In general, none of these factors has complete penetrance and only the combination of some of them lead to the onset of the disease. In this context, epigenetics, acting as a mediator between genome and environment, provides a mechanistic explanation that might offer unique opportunities to increase our understanding of such disorders.^1, 2^ As a consequence, it is not surprising that a number of epigenetically deregulated genes are emerging, allowing us a first glimpse of the importance of epigenetics in neurodegenerative diseases.^3, 4, 5, 6, 7, 8, 9, 10^

The classification of patients into different neurodegenerative disease categories according to the preponderance of symptoms has been important for gaining insight into their pathological hallmarks but it is striking that often no clear distinctions can be made between diseases. Instead, a continuous range of abnormalities is typically observed that, unfortunately, complicates the classification.^11^ The overlap between these diseases could be partly because similar processes are affected in all the disorders. For example, considering the classic pathological hallmarks, we observe that amyloid plaques, which are one of main characteristics of Alzheimer's disease (AD), are also more frequent in dementia with Lewy bodies (DLB) and the early onset of Alzheimer-like neurodegenerative profile associated with Down's syndrome (DS); and that the accumulation of normally soluble proteins into filamentous insoluble aggregates, such as TAU-neurotangles, which are traditional hallmarks of AD and DS, are also found in Parkinson's disease (PD) and DLB. Similarly, characteristic hallmarks of PD and DLB, such as α-synuclein inclusions, are also found in many cases of AD and DS.^12, 13, 14, 15, 16, 17^ There is multiple molecular crossover between these pathways; for example, the α-synuclein protein interacts with TAU, inducing its phosphorylation and aggregation while, simultaneously, TAU enhances α-synuclein aggregation.^18^

To determine whether these neurodegenerative disorders share a common epigenomic defect we obtained the complete human DNA methylomes of the prefrontal cortex of AD, DLB, PD and DS patients at base resolution using whole-genome bisulfite sequencing (WGBS).^19, 20, 21^ We have recently successfully used this method to determine the DNA methylation patterns during the development of the human and mouse brain.^19^ Combining these findings with those from comprehensive DNA methylation microarrays,^22, 23^ we have discovered a DNA methylation landscape that, at the level of both CpG and non-CpG methylation, exhibits a similar pattern of epigenomic disruption for all the neurodegenerative diseases studied here.

## Materials and Methods

### Human samples

Post-mortem tissues were obtained from the Institute of Neuropathology Brain Bank (HUB-ICO-IDIBELL Biobank) following the practice and expertise of BrainNet Europe Bank (http://www.brainnet-europe.org/) ‘Network of Excellence' funded by the European Commission in the sixth Framework Program ‘Life Science' (LSHM-CT-2004-503039). All samples were obtained in agreement with ethical standards and legislation defined by the European Union and following the approval of the local ethics committee. DNA was extracted from the gray matter of the dorsolateral prefrontal cortex (Brodmann area 9). Previously reported WGBS gray and white matter data from the same control donor were used (female, 64 years old; Lister *et al.*^21^). Gray matter from AD (female, 81 years old), DS with AD (male, 41 years old), DLB (female, 77 years old) and PD (female, 77 years old) was extracted for WGBS. Five gray matter samples for each group were hybridized on 450K arrays, and 32 control (9±1 h postmortem interval; aged 66.8±12.0 years), 32 AD (8±1 h postmortem interval; aged 81.6±14.7 years), 5 DS–AD (4±1 h postmortem interval; aged 50.8±9.1 years), 23 DLB (8±1 h postmortem interval; aged 75.5±13.6 years) and 15 PD (7±1 h postmortem interval; aged 72.9±13.1 years) samples were used for pyrosequencing validation.

### Pyrosequencing

The set of primers for PCR amplification and sequencing was designed using the software PyroMark assay design version 2.0.01.15 (Qiagen, Valencia, CA, USA); amplification primers hybridize with CpG-free sites to ensure a methylation-independent reaction, and one primer (opposite the sequencing primer) is biotinylated to convert the PCR product to single-stranded DNA templates. We used 10 ng of bisulfite-treated DNA for each PCR. We used the Vacuum Prep Tool (Qiagen) to prepare single-stranded PCR products according to the manufacturer's instructions. Pyrosequencing reactions and methylation quantification were performed in a PyroMark Q24 System version 2.0.6 (Qiagen). Primers are available upon request.

### Whole-genome bisulfite sequencing data processing

We performed sequence alignment and methylation calling with Bismark v.0.7.4 software (Cambridge, UK). We used hg19 as the reference genome and retrieved genomic information from Biomart (Mansfield, UK). SAM/BAM and BED file-handling was done using SAMtools, bedtools and Tabix. Statistical analysis and graphic representation was carried out with R (http://www.R-project.org) and the multicore and ggplot2 libraries. We defined the promoter region as 2 kb flanking the transcription start site, which was considered to be the most upstream base of all the transcripts of the gene. When depicting methylation profiles, we used the previously described smoothing method.

### CpG distance correlation

We assessed distance correlation of CpG in close proximity, gathering information about methylation and relative distance up to 2000 bases away from all CpG sites, and correlating pair-wise methylation at single CpG sites for each relative distance.

### Detection of DMRs

Differentially methylated regions (DMRs) were identified by seeking regions with more than five consecutive CpG sites consistently located outside the 95% confidence interval of the smoothed methylation profile.

### Detection of histone marks

We obtained human histone marks for human adult brain inferior temporal lobe available from the ENCODE project (GSE17312) and CCAT peak caller, using the standard parameter for histone marks.

### Microarray-based DNA methylation analysis with the Infinium 450K array

The Infinium DNA methylation microarray (Illumina, San Diego, CA, USA) assay was performed as previously previously.^22, 23^

## Results

### WGBS of DNA from gray matter and neurodegenerative disorders

Initial data were generated from the gray matter of a control sample (G145) (64-year-old female Caucasian), and samples of AD (81-year-old female Caucasian), DLB (77-year-old female Caucasian), PD (77-year-old female Caucasian) and neurodegeneration associated with DS (49-year-old male Caucasian) (Table 1), using DNA extracted from the dorsolateral prefrontal cortex Brodmann area 9, because this is particularly involved in higher cognitive skills, such as memory and cognition, and is affected in these neurodegenerative disorders.^24^

**Figure 1:**
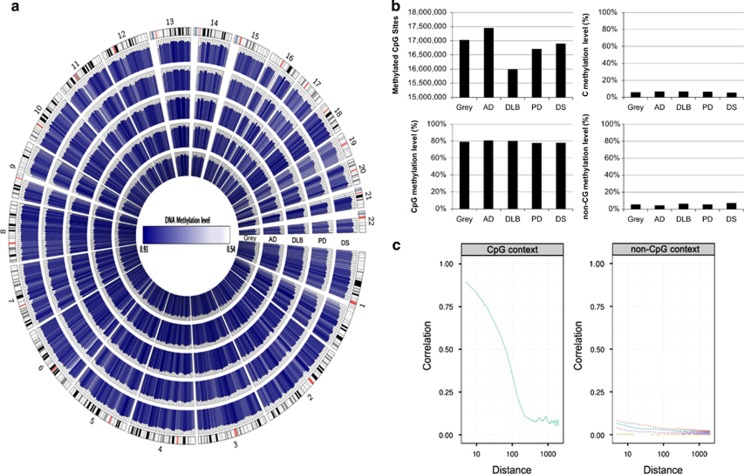
Whole-genome bisulfite sequencing of neurodegenerative diseases. (**a**) Circos representation of genome-wide DNA methylation levels in the control gray matter (Gray) and the affected gray matter of Alzheimer's disease (AD), dementia with Lewy bodies (DLB), Parkinson's disease (PD) and Down's syndrome (DS) individuals. Average levels for all of the CGs in 567 5-Mbp-wide windows. Average methylation levels in all of the regions are expressed as *β*-values (0–1) and are colored blue. (**b**) Total number of methylated CpG sites and the percentage CpG methylation level in the DNA from the control gray matter and the neurodegenerative disorders. Percentage 5-methylcytosine and non-CpG methylation levels are also shown. (**c**) Correlation of methylation status vs distance between dinucleotides (on log scale) in both CpG context (left panel) and non-CpG context (right panel) for the control gray matter sample and the neurodegenerative disorders samples determined by WGBS. We present non-CpG context aggregated (blue line) and broken by dinucleotide (CA, red; CC, brown; CT, pink). A more pronounced declining curve indicates a lower correlation in terms of the methylation status of nearby CpGs.

For WGBS, we generated ~20 × 10^7^ 80–90-bp paired-end reads corresponding to a global coverage of ~10 × sequencing depth. We successfully mapped ~94% of the genome for the studied samples. WGBS coverage and number of reads for each sequenced gray matter case are summarized in Table 1. For all subsequent analyses, to avoid possible gender-related differences, the DNA methylation values derived from the X-chromosome were not included, although other differences in autosomes cannot be completely discarded. The full WGBS data set from the studied gray matter samples is illustrated in Figure 1a, using Circos.^25^ The total number of methylated CpG dinucleotides and the methylcytosine, methyl CpG and non-methyl CpG levels of the samples are shown in Figure 1b. The average methylation level of all CpG sites was similar in the normal and disease-affected gray matter sample, with values ranging from 77.7 to 80.6%. The average methylation level of cytosines in non-CpG sites for all the analyzed samples ranged from 4.3 to 7.3% (Figure 1b). These non-CpG methylation values were similar to those previously reported in brain tissues.^19^ We also observed a correlation in terms of the methylation status of nearby CpGs, which decreases with distance, as we had observed in other tissues,^20^ and showed similar profiles in all samples (Figure 1c). In contrast, no correlation was observed for non-CG methylation (Figure 1c), probably because non-CG methylation is more scattered throughout the genome.^19, 26^ Owing to its proven functional significance and abundance,^27^ we focused subsequent analyses on CpG methylation modification.

### Identification of DMRs

**Figure 2:**
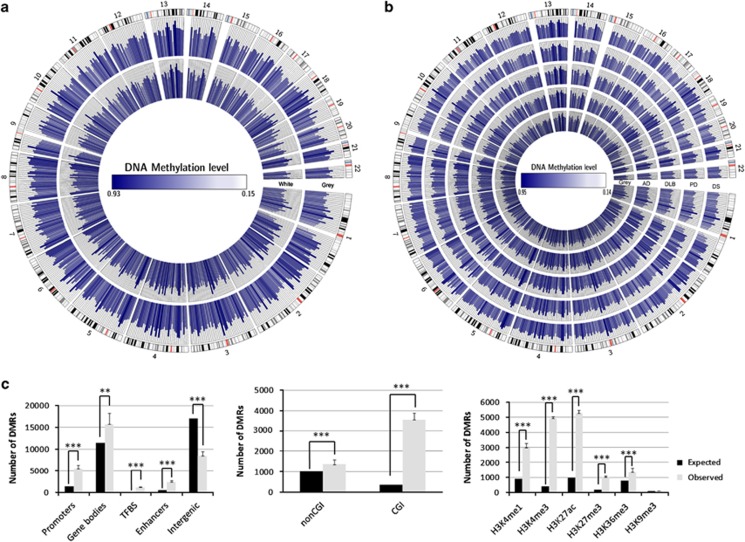
Differentially methylated regions (DMRs) of neurodegenerative diseases. (**a**) Circos representation of average methylation levels for all of the CGs in the DMRs between gray and white matters from the prefrontal cortex of the same person. Regions are equally spaced around the figure and their original locations in the genome are indicated by gray lines. (**b**) Circos representation of average methylation levels for all of the CGs in the DMRs between control gray matter and the affected gray matter of the studied neurodegenerative disorders. Regions are equally spaced around the figure and their original locations in the genome are indicated by gray lines. (**c**) Molecular context of the identified DMRs in neurodegenerative diseases. DMR distribution among different genomic sequences; DMR distribution among promoters with respect to the presence or absence of a CpG island; DMR distribution among different post-translational histone modification marks. *P<0.05; **P<0.01; ***P<0.0001 in Fisher's exact test.

To characterize genomic sequences with a distinct CpG methylation status in the control gray matter and the neurodegenerative diseases under study, we searched for DMRs in the WGBS data using smoothing algorithms.^28^ We thereby identified a range of 25 023–29 817 DMRs between the normal gray matter and the neurodegenerative disorders that might reflect disease-associated and inter-individual differences. DMRs may be associated with distinct percentages of cell types in neurodegenerative diseases where there is a decrease in the number of neurons and an increase in the glial component. To avoid this troublesome possibility in our samples, we performed WGBS in the white matter (W145) of the brain sample in which we had characterized the gray matter component (64-year-old female Caucasian). We identified 26 914 DMRs that distinguished the gray and white matters (G145 vs W145; Figure 2a). We excluded these sequences from our analyses and obtained a range of 21 501–26 140 DMRs in the control gray matter and the neurodegenerative disorder samples. These DMRs are illustrated in Figure 2b using Circos.^25^

The DMRs associated with the neurodegenerative diseases were present particularly in functionally relevant genomic sequences (odds ratio (OR)=2.4; bootstrap *P*<0.001), such as promoters (OR=5.1; bootstrap *P*<0.001), transcription factor-binding sites (OR=8.4; bootstrap *P*<0.001) and enhancers (OR=4.1; bootstrap *P*<0.001; Figure 2c). This observation was particularly striking in promoters located in CpG islands (OR=10.1; bootstrap *P*<0.001), sequences that are key critical regulators of gene expression in relation to their methylation status (Figure 2).

In line with the direct regulatory function of DNA methylation in promoter regions, we found 14–18% (775–930 genes) of differentially methylated genes to be associated with a change in gene expression using gene expression microarray technology and matching RNA from the previously profiled DNA samples (>1.5-fold expression change; Supplementary Tables S1–S4). In particular, we identified 160–363 hypermethylated gene promoters as being related to transcriptional repression (20–42% of differentially expressed genes) and 59–112 hypomethylated promoters as being associated with gene activation (8–14% of differentially expressed genes; Supplementary Tables S1–S4). Most importantly, among these differentially methylated genes with an impact on gene expression in AD (Supplementary Table S1), we confirmed two recently discovered epigenetic targets of the disorder: ankyrin 1 (ANK1) and rhomboid 5 homolog 2 (RHBDF2).^9, 10^ We also further found differential methylation of ANK1 in DLB (Supplementary Table S3).

The functional role of the identified DMRs was also highlighted by their association with another critical layer of epigenetic control, the post-translational modification of histones.^29^ Using the histone mark map for human adult brain inferior temporal lobe available from the ROADMAP project (GSE17312) and the CCAT peak caller,^30^ we observed that >95% of the DMRs co-localized with euchromatin markers such as H3K4me1, H3K4me3, H3K36me3 and H3K27ac (OR=5.7; bootstrap *P*<0.001), while heterochromatin regions remained largely under-represented in our DMR setting (H3K9me3; OR=1.3; bootstrap *P*<0.001; Figure 2d). Furthermore, there was a particular enrichment on bivalent domains (H3K4me3+H3K27me3) in our DMRs (OR=14.6; bootstrap *P*<0.001; Figure 2d), which is an additional indication of a putative functional role.

### A common core of DNA methylation changes underlies neurodegenerative diseases

To gain initial insight into the cellular pathways targeted by those DMRs that overlapped gene promoter genes, we used the gProfiler analysis suite,^31^ which revealed a number of significantly overrepresented KEGG pathways that influence brain development and function (Table 2). These included not only synapse pathways, genes associated with the regulation of cytoskeleton, neurotrophin and ErbB signaling but also developmental networks such as those associated with the Wnt and Hippo pathways (Table 2). The striking overlap of the epigenetically enriched, disrupted pathways between AD, DLB, PD and DS prompted us to examine whether a common core of genes did indeed have altered DNA methylation patterns for all these neurodegenerative disorders.

**Figure 3:**
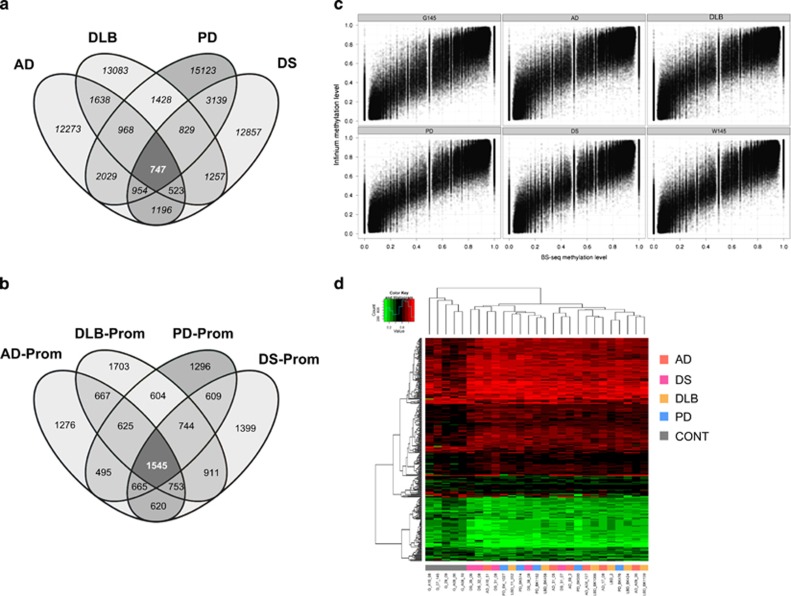
A common set of gene-associated DMRs for neurodegenerative diseases. (**a**) Venn diagram representing the shared DMRs for the neurodegenerative disorders with respect to the DMRs present in overlapping promoter sequences. (**b**) Venn diagram representing the shared DMRs for the neurodegenerative disorders that occurred in the same gene promoters but whose sequences did not overlap. (**c**) Scatterplot comparison of CpG methylation level obtained by WGBS (*x* axis) and the Infinium 450K array (*y* axis) technologies for each analyzed sample. All differentially methylated CpG sites are shown that were identified by the WGBS approach and that were present on the 450K DNA methylation microarray. (**d**) Heatmap clustering of 450K DNA methylation microarray data representing the 747 (left) and 1545 (right) DMRs shared by all four neurodegenerative diseases. Red and green indicate high and low levels of DNA methylation, respectively. Hierarchical clustering by Euclidian distance was carried out. AD, Alzheimer's disease; CONT, control gray matter; DMR, differentially methylated region; DLB; dementia with Lewy bodies; DS, Alzheimer-like neurodegenerative profile associated with Down's syndrome; PD, Parkinson's disease; WGBS, whole-genome bisulfite sequencing.

We adopted a dual approach to assess the commonality of DMRs among the four neurodegenerative diseases. First, following a very stringent criterion, we examined how many of the DMRs occurred in overlapping promoter sequences for the characterized disorders. This strategy yielded 709 DMRs that were present in all these neurodegenerative diseases, including 257 differentially methylated gene promoters (Figure 3a and Supplementary Table S5). Second, in a relatively less stringent manner, we assessed how many of the DMRs occurred in the same gene promoters even though their sequences did not overlap. This approach yielded 1545 DMRs that were shared by AD, DLB, PD and neurodegeneration associated with DS (Figure 3a and Supplementary Table S6).

To further validate the DNA methylation signature obtained for the neurodegenerative diseases, we sought to extrapolate our findings from the WGBS data and their corresponding DMR analyses to a larger collection of samples of these disorders. We used an established DNA methylation microarray that assays the DNA methylation status of 450,000 CpG sites.^22, 23^ Although we have already shown that this microarray platform produces accurate DNA methylation data at a similar level than WGBS,^20^ we nevertheless reassessed this finding. The methylation levels obtained from all 450K CpG sites included in the microarray were significantly correlated with those values obtained with the WGBS technology (Pearson correlation, *r*^2^=0.95, *P*<0.01; Figure 3c). As the analysis of single samples impedes comprehensive statistical analysis, we use the technically validated CpG sites located in the 709 and 1545 WGBS-derived DMRs common to all the diseases under consideration for a profound validation in a larger sample cohort using genome-scale analysis (450K). We found significant differences in the DNA methylation patterns between the control gray matter and that derived from an additional set of 20 cases of neurodegenerative disorders (multiscale bootstrap resampling, *n*=1000, *P*<0.0001), which enabled them to be distinguished by hierarchical clustering (Figure 3d).

**Figure 4:**
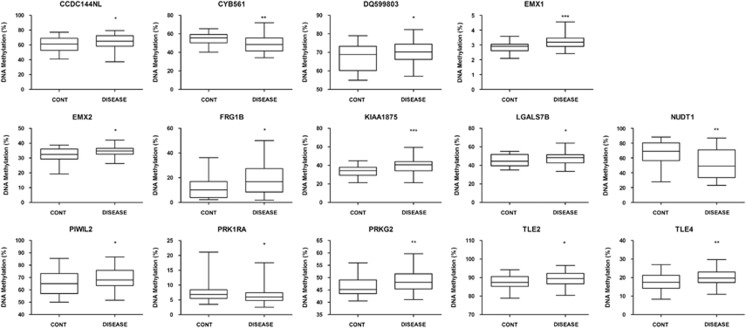
Pyrosequencing validation of candidates genes with a shared CpG methylation shift in all neurodegenerative diseases. The box-plots show the distribution of CpG methylation for each gene in control gray matter and the four neurodegenerative disorders. The central solid line represents the median; the limits of the box indicate the upper and lower quartiles. The whiskers represent the minimum and maximum values, excluding outliers (<1.5 × the interquartile range). *P<0.05; **P<0.01; and ***P<0.001 in Fisher's exact test.

We further validated the differential DNA methylation status of the identified CpG sites by developing a pyrosequencing assay for 20 top-ranked candidates of the WGBS-derived candidate genes identified here (Supplementary Table S6) in an additional set of 32 control and 75 neurodegenerative disease-associated gray matter samples (Figure 4 and Supplementary Table S7). In this setting, we confirmed the significant differential methylation status of these genes in AD, DLB, PD and DS relative to the control gray matter (Figure 4), confirming the aforementioned distinct CpG methylation patterns obtained from different samples and by different techniques (WGBS and 450K DNA methylation microarray).

Overall, these results suggest that the most frequent human neurodegenerative disorders share a number of epigenetic alterations in target genes. The existence of an aberrant DNA methylation landscape that is common to AD, DLB, PD and DS could be used to devise new biomarkers of the diseases, and to explain, in part, their physiopathology. This could provide a scientific basis for the preclinical use of drugs targeting the epigenome of neurodegenerative disorders.

## Discussion

In recent years the importance of DNA methylation in brain functionality has gained increasing recognition.^3, 4, 5, 6, 7, 8, 9, 10^ Several studies, including ours, have focused on the determination of the DNA methylation patterns of neurons and glia, whereas others have concentrated on particular brain regions. Although these have produced interesting findings, the analyses have some limitations. For example, in examining specific regions, the distinct cell-type makeup is often not taken into account, and the pure comparison of the neurons and glial cells does not consider the peculiarities of brain substructures. The two physiological components, cell-type composition and local brain regions, cross talk at the level of activity and it is known that neurons and glial cells have specific spatial profiles associated with the classic anatomical structures involved in particular functions.^32, 33^ Another limitation of many of the previous studies of brain epigenetics also relates to the methods used, whereby only target candidate genes or those sequences included in the available DNA methylation arrays could be interrogated.^3, 4, 5, 6, 7, 8^ To overcome these concerns, in this study we obtained the complete and unbiased DNA methylome by WGBS^19, 20^ of an important and specific brain region—the gray matter from the dorsolateral prefrontal cortex Brodmann area 9 (ref. 24)—from normal status cases and from four prevalent neurodegenerative disorders. We subtracted the DNA methylome of the white matter from the same area to identify the significant DMRs that characterize the disorders.

Two recent global DNA methylation analyses of AD^9, 10^ have provided the first complete DNA methylomes of the disorder. These two independent epigenome-wide studies identified four common aberrantly methylated genes (*ANK1*, *RHBDF2*, *RPL13* and *CDH23*). Our WGBS approach enabled us to validate these findings for ANK1 and RHBDF2 in AD, and for ANK1 in DLB. In this regard, and to the best of our knowledge, our study represents the first common analysis of the entire DNA methylomes at single-nucleotide resolution of the most frequent neurodegenerative diseases in western societies. In this context, additionally obtaining the DNA methylomes of glial cells from the same area was especially important because a specific loss of neurons occurs in these neurodegenerative diseases that could have yielded false-positive DMRs. Interestingly, we found an outstanding coincidence of the DNA methylation alterations and their associated cellular pathways present in the four neurodegenerative disorders studied here. Thus, a common substrate of epigenetic defects underlies these diseases, and this could help explain some of their shared molecular, cellular and clinical features. In this regard, more than 50% of AD people show α-synuclein inclusions, whereas comorbid AD pathologies, including β-amyloid plaques and neurotangles, are commonly found in PD and DLB brains,^15^ and α-synuclein inclusions are also frequently found in DS.^12^ It has also been observed that familial forms of AD, harboring APP, PSEN1 or PSEN2 mutations, present α-synuclein inclusions in more than 60% of cases, and that PD patients harboring the dominant mutation A53T SNCA have a higher prevalence of dementia than those with idiopathic forms.^15^ Furthermore, the incidence of dementia in PD patients is up to six times that observed in normal people.^15^ Therefore, our data are consistent with the aforementioned observations, and many others, that reinforce the idea that these neurodegenerative diseases might represent a wide spectrum of manifestations of pathologies with similar causative mechanisms.

Although we cannot discard that our initially produced WGBS data might represent a combination of disease-associated and inter-individual differences, the subsequent thorough validation steps performed in this study strongly supported the biological meaning of our observations. In particular, following stringent filtering of the initial set of DMRs for cell-type composition, their enrichment in functional genomic elements largely excludes randomness of the identified loci. Importantly, the limitation of small initial sample sizes was subsequently addressed combining validation steps of larger sample cohorts at genome-scale and loci-specific allowing a rigorous statistical analysis to conform the significance of the identified differential methylated loci. Strikingly, considering the limited initial sample size and the small absolute differences in DNA methylation between the diseases, we could validate commonly differentially methylated loci throughout the validation cohorts. This is of particular importance as our approach could influence the design of future epigenome-wide association studies in neurodegenerative diseases. In contrary to genome-wide association study, epigenetic studies might present significant results already for smaller cohorts, due to the less random character of DNA methylation changes that are likely to be directly driven by the disease pathology. Considering the flexible character of epigenetic marks, influenced by aging processes or environmental effects, it is important to widely exclude confounding factor, such as age, gender or origin, through a balanced sample design (as performed in the here-presented study, Supplementary Table S7). It is of note that our analysis of samples from both genders in a combined manner could miss important variance within the gender groups (sex chromosomes), which has to be addressed in future work using larger gender-matched cohorts.

Further clues to the origin of these disorders can be found in the pathways represented by the genes associated with the DMRs characterized in our study. For example, we found that key genes for brain development and cerebral cortex regionalization, such as the Empty Spiracle Homeobox 1 and 2 (*EMX1* and *EMX2*) genes,^34^ were aberrantly methylated in the neurodegenerative disorders. These findings are linked to the recent observation that amyloid peptide treatments can induce DNA methylation alterations in genes involved in *in vitro* neuronal differentiation.^35^ Other pathways associated with neurodegenerative diseases, such as altered cyclic nucleotide signaling,^36^ illustrated by the protein kinase cAMP-dependent regulatory type I alpha (*PRKAR1A*) and the cGMP-dependent protein kinase type II (*PRKG2*) genes, were also present in the identified DMRs. Interestingly, the characterized CpG methylation sites might also pinpoint newly recognized pathways in these disorders. This would include, for example, the contribution of other epigenetic marks, such as histone modifications related to the transducin-like enhancers of split 2 and split 4 proteins, which are both partners of the histone deacetylase 1 complex that has been linked to neurodegeneration.^37^ It would also include the first-in-class description of the disruption of DNA methylation by a non-coding RNA (DQ599803) that is transcribed from a piRNA genomic locus, a family of small non-coding RNAs that are essential for neuronal plasticity and memory formation,^38^ and that are involved in TAU-mediated neurodegeneration.^39^ Further intense *in vitro* and *in vivo* work will be required in the coming years to gather further evidence of the role of these candidate genes in the origin and progression of these diseases.

In conclusion, we present the DNA methylation patterns of the most common neurodegenerative disorders at the highest level of resolution reported to date. The picture that emerges indicates that AD, DLB, PD and DS share an aberrant DNA methylation signature characterized by the presence of the disrupted CpG methylation status of a set of common genes involved in many cellular pathways. These findings suggest that the disorders might have similar early pathogenetic mechanisms that subsequently evolve into clinical entities with different molecular and cellular features. The publicly deposited DNA methylation data obtained here are important as reference epigenomes for these disorders, and could be further explored using other bioinformatic algorithms to derive additional relevant information about these diseases. Although much effort will be required in the near future to disentangle the functional role of some of the observed changes, our results indicate that a distorted DNA methylation landscape is a hallmark of human neurodegeneration.

## Figures and Tables

**Table 1:** WGBS samples and general data summary

*Sample description*						*Deep sequencing data*	
*Code*	*Neurodegenerative disease*	*Region*	*Age*	*Gender*	*Individual group*	*Coverage*	*No. of reads*
A09	Alzheimer's disease	BA9 gray matter	81	Female	Caucasian	10.8 ×	193 166 927
DLB2	Dementia with Lewy bodies	BA9 gray matter	77	Female	Caucasian	8.3 ×	150 659 414
BK1027	Parkinson's disease	BA9 gray matter	77	Female	Caucasian	12.1 ×	231 693 584
31_08	Down syndrome with Alzheimer's disease	BA9 gray matter	49	Male	Caucasian	11.0 ×	199 508 612
G145	Control gray matter	BA9 gray matter	64	Female	Caucasian	10.6 ×	190 679 672
W145	Control white matter	BA9 white matter	64	Female	Caucasian	10.7 ×	191 543 442

Abbreviations: BA9, Brodmann area 9; WGBS, whole-genome bisulfite sequencing.

**Table 2:** KEGG analysis of differentially methylated promoters

*Term ID*	*Description*	*Diseases*	*AD* P*-value*	*DS* P*-value*	*DLB* P*-value*	*DS* P*-value*
KEGG:04012	ErbB signaling pathway	AD, DS, DLB and PD	0.0000387	0.0237	0.000331	0.00229
KEGG:04350	TGF-beta signaling pathway	AD, DS, DLB and PD	0.0372	0.0237	0.0362	0.0326
KEGG:04810	Regulation of actin cytoskeleton	AD, DS, DLB and PD	0.00191	0.0000792	0.0000258	0.0151
KEGG:04390	Hippo signaling pathway	AD, DS, DLB and PD	0.00000599	3.17E-10	0.000187	0.0000209
KEGG:04310	Wnt signaling pathway	AD, DS, DLB and PD	1.37E-07	0.000512	0.00011	0.0251
KEGG:04010	MAPK signaling pathway	AD, DS, DLBD and PD	0.000156	0.0334	0.00000113	0.0000373
KEGG:04722	Neurotrophin signaling pathway	AD, DS, DLB and PD	3.09E-07	0.0182	0.0000206	0.00000195
KEGG:04666	Fc gamma R-mediated phagocytosis	AD, DS, DLB and PD	0.0000548	0.00623	0.000414	0.00738
KEGG:04115	p53 signaling pathway	AD, DS, DLBD and PD	0.00052	0.0262	0.033	0.0000254
KEGG:04520	Adherens junction	AD, DS, DLB and PD	0.000516	0.0299	0.00143	0.0197
KEGG:04340	Hedgehog signaling pathway	AD, DS and DLB	0.032	0.0109	0.00309	—
KEGG:04725	Cholinergic synapse	AD, DLB and PD	0.00341	—	0.0033	0.0106
KEGG:04110	Cell cycle	AD, DS and PD	0.0000282	0.045	—	1.15E-07
KEGG:04724	Glutamatergic synapse	AD and DLB	0.000733	—	0.0395	—
KEGG:04713	Circadian entrainment	AD and PD	0.00286	—	—	0.0376
KEGG:04728	Dopaminergic synapse	AD and PD	0.0339	—	—	0.03
KEGG:04360	Axon guidance	DS and PD	—	0.000119	—	0.0198
KEGG:04120	Ubiquitin-mediated proteolysis	DS and PD	—	0.0103	—	0.00156
KEGG:04151	PI3K–Akt signaling pathway	DLB and PD	—	—	0.000028	0.00772
KEGG:04070	Phosphatidyl inositol signaling system	DLB and PD	—	—	0.00198	0.0016
KEGG:04150	mTOR signaling pathway	DLB and PD	—	—	0.0181	0.00127
KEGG:04710	Circadian rhythm	AD	0.0411	—	—	—
KEGG:00532	Glycosaminoglycan biosynthesis—chondroitin sulfate/dermatan sulfate	AD	0.00626	—	—	—
KEGG:04066	HIF-1 signaling pathway	AD	—	—	—	—
KEGG:04530	Tight junction	DS	—	0.0158	—	—
KEGG:00562	Inositol phosphate metabolism	DLB	—	—	0.000316	—
KEGG:04510	Focal adhesion	DLB	—	—	0.000525	—
KEGG:04910	Insulin signaling pathway	PD	—	—	—	0.0149
KEGG:04270	Vascular smooth muscle contraction	PD	—	—	—	0.0339

Abbreviations: AD, Alzheimer's disease; DLB, dementia with Lewy bodies; DS, Down's syndrome; HIF-1, hypoxia-inducible factor-1; MAPK, mitogen-activated protein kinase; PD, Parkinson's disease; TGF, transforming growth factor.
