# Supplementary material for: Human DNA methylomes of neurodegenerative diseases show common epigenomic patterns
Source: Transl Psychiatry. 2016 Jan 19;6(1):e718–. doi: 10.1038/tp.2015.214 (PMC5068885; doi:10.1038/tp.2015.214)
Supplement: Supplementary Table 7 [file tp2015214x7.docx]

**Table S7:** Clinicopathological data of samples included in the study.

| Sample | Status | Brain Region | Braak Staging | PMI | Age | Gender | Analysed by |
| --- | --- | --- | --- | --- | --- | --- | --- |
| CONT _1 | Normal | Frontal Cortex | 0 | 9:35 | 67 | F | WGBS, 450K, Pyrosequencing |
| CONT _2 | Control | Frontal Cortex | II A | 2:00 | 83 | F | 450K, pyrosequencing |
| CONT _3 | Normal | Frontal Cortex | 0 | 3:40 | 64 | M | 450K, pyrosequencing |
| CONT _4 | Normal | Frontal Cortex | I | 15:45 | 73 | F | 450K, pyrosequencing |
| CONT _5 | Normal | Frontal Cortex | 0 | 8:05 | 53 | M | 450K, pyrosequencing |
| CONT _6 | Normal | Frontal Cortex | 0 | 13:30 | 37 | F | Pyrosequencing |
| CONT _7 | Normal | Frontal Cortex | I | 6:00 | 79 | M | Pyrosequencing |
| CONT _8 | Normal | Frontal Cortex | 0 | 14:00 | 70 | M | Pyrosequencing |
| CONT _9 | Normal | Frontal Cortex | I | 13:00 | 68 | F | Pyrosequencing |
| CONT _10 | Normal | Frontal Cortex | 0 | 14:05 | 46 | F | Pyrosequencing |
| CONT _11 | Normal | Frontal Cortex | 0 | 4:15 | 84 | F | Pyrosequencing |
| CONT _12 | Normal | Frontal Cortex | I | 14:00 | 78 | F | Pyrosequencing |
| CONT _13 | Normal | Frontal Cortex | II | 5:15 | 83 | F | Pyrosequencing |
| CONT _14 | Normal | Frontal Cortex | 0 | 3:35 | 69 | M | Pyrosequencing |
| CONT _15 | Normal | Frontal Cortex | II | 15:00 | 79 | F | Pyrosequencing |
| CONT _16 | Normal | Frontal Cortex | 0 | 4:35 | 56 | M | Pyrosequencing |
| CONT _17 | Normal | Frontal Cortex | I | 14:40 | 67 | M | Pyrosequencing |
| CONT _18 | Normal | Frontal Cortex | 0 | 10:45 | 58 | M | Pyrosequencing |
| CONT _19 | Normal | Frontal Cortex | I | 9:55 | 63 | M | Pyrosequencing |
| CONT _20 | Normal | Frontal Cortex | 0 | 8:30 | 55 | M | Pyrosequencing |
| CONT _21 | Normal | Frontal Cortex | 0 | 7:00 | 61 | F | Pyrosequencing |
| CONT _22 | Normal | Frontal Cortex | I | 3:30 | 69 | M | Pyrosequencing |
| CONT _23 | Normal | Frontal Cortex | I | 5:00 | 78 | M | Pyrosequencing |
| CONT _24 | Normal | Frontal Cortex | 0 | 2:15 | 64 | F | Pyrosequencing |
| CONT _25 | Normal | Frontal Cortex | II | 4:20 | 74 | M | Pyrosequencing |
| CONT _26 | Normal | Frontal Cortex | 0 | 11:25 | 56 | M | Pyrosequencing |
| CONT _27 | Normal | Frontal Cortex | II | 3:30 | 79 | F | Pyrosequencing |
| CONT _28 | Normal | Frontal Cortex | 0 | 6:00 | 61 | M | Pyrosequencing |
| CONT _29 | Normal | Frontal Cortex | II | 20:00 | 75 | F | Pyrosequencing |
| CONT _30 | Normal | Frontal Cortex | I | 16:00 | 67 | F | Pyrosequencing |
| CONT _31 | Normal | Frontal Cortex | 0 | 13:30 | 47 | F | Pyrosequencing |
| CONT _32 | Normal | Frontal Cortex | I | 18:00 | 71 | F | Pyrosequencing |
| CONT _33 | Normal | Frontal Cortex | II | 17:30 | 71 | M | Pyrosequencing |
| AD_1 | Alzheimer Disease | Frontal Cortex | V C | 6:30 | 81 | F | WGBS, 450K, Pyrosequencing |
| AD_2 | Alzheimer Disease | Frontal Cortex | VI C | 6:00 | 79 | M | 450K, pyrosequencing |
| AD_3 | Alzheimer Disease | Frontal Cortex | V C | 8:30 | 84 | F | 450K, pyrosequencing |
| AD_4 | Alzheimer Disease | Frontal Cortex | V C | 16:15 | 85 | F | 450K, pyrosequencing |
| AD_5 | Alzheimer Disease | Frontal Cortex | V C | 7:05 | 87 | M | 450K, pyrosequencing |
| AD_6 | Alzheimer Disease | Frontal Cortex | V C | 7:50 | 92 | M | Pyrosequencing |
| AD_7 | Alzheimer Disease | Frontal Cortex | VI C | 5:30 | 75 | F | Pyrosequencing |
| AD_8 | Alzheimer Disease | Frontal Cortex | V C | 7:30 | 94 | F | Pyrosequencing |
| AD_9 | Alzheimer Disease | Frontal Cortex | V C | 8:00 | 63 | M | Pyrosequencing |
| AD_10 | Alzheimer Disease | Frontal Cortex | V C | 9:15 | 78 | F | Pyrosequencing |
| AD_11 | Alzheimer Disease | Frontal Cortex | VI C | 13:50 | 63 | F | Pyrosequencing |
| AD_12 | Alzheimer Disease | Frontal Cortex | V C | 16:00 | 77 | M | Pyrosequencing |
| AD_13 | Alzheimer Disease | Frontal Cortex | VI C | 4:00 | 98 | F | Pyrosequencing |
| AD_14 | Alzheimer Disease | Frontal Cortex | VI C | 5:00 | 75 | M | Pyrosequencing |
| AD_15 | Alzheimer Disease | Frontal Cortex | VI C | 5:30 | 88 | F | Pyrosequencing |
| AD_16 | Alzheimer Disease | Frontal Cortex | VI C | 6:00 | 79 | F | Pyrosequencing |
| AD_17 | Alzheimer Disease | Frontal Cortex | V C | 6:15 | 87 | F | Pyrosequencing |
| AD_18 | Alzheimer Disease | Frontal Cortex | VI C | 5:30 | 97 | F | Pyrosequencing |
| AD_19 | Alzheimer Disease | Frontal Cortex | V C | 4:40 | 92 | F | Pyrosequencing |
| AD_20 | Alzheimer Disease | Frontal Cortex | VI C | 8:00 | 67 | F | Pyrosequencing |
| AD_21 | Alzheimer Disease | Frontal Cortex | V B | 5:25 | 69 | M | Pyrosequencing |
| AD_22 | Alzheimer Disease | Frontal Cortex | V B | 22:15 | 76 | M | Pyrosequencing |
| AD_23 | Alzheimer Disease | Frontal Cortex | V C | 5:30 | 94 | F | Pyrosequencing |
| AD_24 | Alzheimer Disease | Frontal Cortex | V C | 9:10 | 86 | F | Pyrosequencing |
| AD_25 | Alzheimer Disease | Frontal Cortex | V C | 8:45 | 72 | M | Pyrosequencing |
| AD_26 | Alzheimer Disease | Frontal Cortex | VI C | 20:35 | 86 | F | Pyrosequencing |
| AD_27 | Alzheimer Disease | Frontal Cortex | V C | 5:20 | 79 | F | Pyrosequencing |
| AD_28 | Alzheimer Disease | Frontal Cortex | V C | 2:00 | 83 | M | Pyrosequencing |
| AD_29 | Alzheimer Disease | Frontal Cortex | VI C | 6:40 | 78 | M | Pyrosequencing |
| AD_30 | Alzheimer Disease | Frontal Cortex | V C | 11:30 | 76 | F | Pyrosequencing |
| AD_31 | Alzheimer Disease | Frontal Cortex | V B | 5:30 | 86 | F | Pyrosequencing |
| AD_32 | Alzheimer Disease | Frontal Cortex | V C | 7:00 | 83 | M | Pyrosequencing |
| AD_33 | Alzheimer Disease | Frontal Cortex | V C | 14:25 | 84 | F | Pyrosequencing |
| DS_01 | Down's Syndrome | Frontal Cortex | VI C | 2:12 | 49 | M | WGBS, 450K, Pyrosequencing |
| DS_02 | Down's Syndrome | Frontal Cortex | VI C | 4:37 | 52 | F | 450K, pyrosequencing |
| DS_03 | Down's Syndrome | Frontal Cortex | V C | 5:00 | 42 | F | 450K, pyrosequencing |
| DS_04 | Down's Syndrome | Frontal Cortex | III B | 9:00 | 48 | F | 450K, pyrosequencing |
| DS_05 | Down's Syndrome | Frontal Cortex | VI C | 3:00 | 63 | F | 450K, pyrosequencing |
| DLB_01 | Dementia with Lewy Bodies | Frontal Cortex | DLB Neocortical | 5:45 | 77 | F | WGBS, 450K, Pyrosequencing |
| DLB_02 | Dementia with Lewy Bodies | Frontal Cortex | DLB Neocortical | 7:45 | 79 | M | 450K, pyrosequencing |
| DLB_03 | Dementia with Lewy Bodies | Frontal Cortex | DLB Neocortical | 6:00 | 71 | F | 450K, pyrosequencing |
| DLB_04 | Dementia with Lewy Bodies | Frontal Cortex | DLB Neocortical | 16:30 | 81 | M | 450K, pyrosequencing |
| DLB_05 | Dementia with Lewy Bodies | Frontal Cortex | DLB Neocortical | 11:00 | 77 | M | 450K, pyrosequencing |
| DLB_06 | Dementia with Lewy Bodies | Frontal Cortex | DLB Neocortical | 9:00 | 85 | F | Pyrosequencing |
| DLB_07 | Dementia with Lewy Bodies | Frontal Cortex | DLB Neocortical | 12:00 | 68 | M | Pyrosequencing |
| DLB_08 | Dementia with Lewy Bodies | Frontal Cortex | DLB Neocortical | 3:30 | 72 | F | Pyrosequencing |
| DLB_09 | Dementia with Lewy Bodies | Frontal Cortex | DLB Neocortical | 8:00 | 60 | M | Pyrosequencing |
| DLB_10 | Dementia with Lewy Bodies | Frontal Cortex | DLB Neocortical | 8:15 | 64 | M | Pyrosequencing |
| DLB_11 | Dementia with Lewy Bodies | Frontal Cortex | DLB Neocortical | 7:45 | 72 | M | Pyrosequencing |
| DLB_12 | Dementia with Lewy Bodies | Frontal Cortex | DLB Limbic | 6:00 | 87 | F | Pyrosequencing |
| DLB_13 | Dementia with Lewy Bodies | Frontal Cortex | DLB Neocortical | 8:15 | 71 | F | Pyrosequencing |
| DLB_14 | Dementia with Lewy Bodies | Frontal Cortex | DLB Neocortical | 7:20 | 76 | M | Pyrosequencing |
| DLB_15 | Dementia with Lewy Bodies | Frontal Cortex | DLB Neocortical | 3:15 | 83 | M | Pyrosequencing |
| DLB_16 | Dementia with Lewy Bodies | Frontal Cortex | DLB Neocortical | 8:00 | 80 | M | Pyrosequencing |
| DLB_17 | Dementia with Lewy Bodies | Frontal Cortex | DLB Neocortical | 5:00 | 64 | M | Pyrosequencing |
| DLB_18 | Dementia with Lewy Bodies | Frontal Cortex | DLB Neocortical | 14:20 | 72 | M | Pyrosequencing |
| DLB_19 | Dementia with Lewy Bodies | Frontal Cortex | DLB Neocortical | 7:00 | 80 | M | Pyrosequencing |
| DLB_20 | Dementia with Lewy Bodies | Frontal Cortex | DLB Neocortical | 6:45 | 77 | F | Pyrosequencing |
| DLB_21 | Dementia with Lewy Bodies | Frontal Cortex | DLB Neocortical | 4:30 | 77 | F | Pyrosequencing |
| DLB_22 | Dementia with Lewy Bodies | Frontal Cortex | DLB Neocortical | 6:20 | 82 | F | Pyrosequencing |
| DLB_23 | Dementia with Lewy Bodies | Frontal Cortex | DLB Neocortical | 5:00 | 81 | M | Pyrosequencing |
| PD_01 | Parkinson Disease | Frontal Cortex | VI | 5:45 | 77 | F | WGBS, 450K, Pyrosequencing |
| PD_02 | Parkinson Disease | Frontal Cortex | IV | 7:45 | 72 | M | 450K, pyrosequencing |
| PD_03 | Parkinson Disease | Frontal Cortex | IV | 3:30 | 79 | F | 450K, pyrosequencing |
| PD_04 | Parkinson Disease | Frontal Cortex | IV | 9:20 | 68 | M | 450K, pyrosequencing |
| PD_05 | Parkinson Disease | Frontal Cortex | V | 5:30 | 71 | M | 450K, pyrosequencing |
| PD_06 | Parkinson Disease | Frontal Cortex | V | 8:30 | 70 | F | Pyrosequencing |
| PD_07 | Parkinson Disease | Frontal Cortex | IV | 4:30 | 69 | F | Pyrosequencing |
| PD_08 | Parkinson Disease | Frontal Cortex | V | 3:30 | 73 | M | Pyrosequencing |
| PD_09 | Parkinson Disease | Frontal Cortex | IV | 5:30 | 74 | M | Pyrosequencing |
| PD_10 | Parkinson Disease | Frontal Cortex | V | 7:30 | 77 | F | Pyrosequencing |
| PD_11 | Parkinson Disease | Frontal Cortex | VI | 7:30 | 80 | M | Pyrosequencing |
| PD_12 | Parkinson Disease | Frontal Cortex | IV | 4:30 | 84 | F | Pyrosequencing |
| PD_13 | Parkinson Disease | Frontal Cortex | V | 7:30 | 80 | M | Pyrosequencing |
| PD_14 | Parkinson Disease | Frontal Cortex | V | 6:10 | 69 | F | Pyrosequencing |
| PD_15 | Parkinson Disease | Frontal Cortex | V | 16:30 | 50 | M | Pyrosequencing |
